# Event boundaries do not cause the immediate extinction deficit after Pavlovian fear conditioning in rats

**DOI:** 10.1038/s41598-019-46010-4

**Published:** 2019-07-01

**Authors:** Michael S. Totty, Martin R. Payne, Stephen Maren

**Affiliations:** Department of Psychological and Brain Sciences and Institute for Neuroscience, Texas A&M University, College Station, Texas 77843 USA

**Keywords:** Extinction, Fear conditioning

## Abstract

Recent work reveals that the extinction of conditioned fear depends upon the interval between conditioning and extinction. Extinction training that takes place within minutes to hours after fear conditioning fails to produce a long-term extinction memory, a phenomenon known as the immediate extinction deficit (IED). Neurobiological evidence suggests that the IED results from stress-induced dysregulation of prefrontal cortical circuits involved in extinction learning. However, a recent study in humans suggests that an “event boundary” between fear conditioning and extinction protects the conditioning memory from interference by the extinction memory, resulting in high levels of fear during a retrieval test. Here, we contrast these hypotheses in rats by arranging extinction trials to follow conditioning trials with or without an event boundary; in both cases, extinction trials are delivered in proximity to shock-elicited stress. After fear conditioning, rats either received extinction trials 60-sec after the last conditioning trial (continuous, no event boundary) or 15-minutes after conditioning (segmented, a standard “immediate” extinction procedure associated with an event boundary). Both groups of animals showed decreases in conditional freezing to the auditory conditioned stimulus (CS) during extinction and exhibited an equivalent IED relative to non-extinguished controls when tested 48 hours later. Thus, eliminating the event boundary between conditioning and extinction with the continuous extinction procedure did not prevent the IED. These data suggest that the IED is the result of shock-induced stress, rather than boundary-induced reductions in memory interference.

## Introduction

Traumatic events often result in long-lasting emotional memories that persist for years. Commonly used therapeutic strategies for pathological fear include exposure therapy, a procedure in which reminders of the trauma are presented in a safe context in order to reduce maladaptive fear. Extinction procedures, such as exposure therapy, are thought to work by forming a new inhibitory “extinction” memory that interferes with the expression of the original “fear” memory. However, there are numerous circumstances by which extinction learning fails to result in a long-term extinction memory. Recent data from both humans and rodents suggest that extinction training that takes place soon (~15 min–6 hours) after fear conditioning fails to reduce fear to the extinguished cue when tested days (48 hr) later; a phenomenon that has come to be known as the immediate extinction deficit (IED)^1,2^.

**Figure 1:**
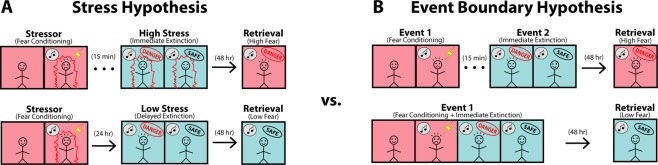
Illustration depicting two competing hypotheses of the nature and cause of the IED. (**A**) We propose that the hyperarousal associated with fear conditioning dysregulates the neural circuits critical for extinction learning, thus resulting in a deficit in extinction procedures that take place immediately following conditioning^3^. Extinction procedures that occur in a low stress state (i.e., delayed extinction or immediate extinction with pharmacological aid) result in diminished fear responses when tested days later. (**B)** Dunsmoor and colleagues^7^ suggest that the ~15 min break normally given between fear conditioning and extinction acts as an event boundary which prioritizes the consolidation of the original fear memory at the expense of the subsequent extinction memory. By this view, a truly continuous extinction procedure (i.e., without an event boundary) allows the extinction memory to interfere with the conditioning memory, thus resulting in diminished fear responses when tested days later. The red and blue background colors represent high and low fear, respectively, and are not representative of different experimental contexts.

Although the underlying mechanism for the IED is not understood, we have proposed that the stress and emotional arousal associated with fear conditioning impedes extinction learning^3^ (Fig. 1A). Consistent with this, the IED is not observed after fear conditioning procedures that use relatively weak shock^4^ or a single conditioning trial^1^. Moreover, delivering arousing footshocks prior to a delayed extinction procedure impairs long-term extinction^1^. It is well known that stress dysregulates the medial prefrontal cortical circuitry involved in extinction learning^5^. We have recently shown that fear conditioning is associated with a reduction in the spontaneous firing of infralimbic (IL) cortical neurons involved in extinction learning; systemic propranolol administration eliminates this effect and rescues the IED^6^. Hence, the suppression of IL activity precipitated by the stress and noradrenergic hyperarousal associated with fear conditioning may be responsible for the IED.

A recent study in humans, however, offers a different interpretation of the IED. As shown in Fig. 1B, Dunsmoor and colleagues^7^ have proposed that a temporal break (an “event boundary”) between conditioning and extinction might protect the conditioning memory from interference by the extinction memory^7^. This process, known as “event segmentation”, is critical for organizing daily events into unique memories that are distinct from previous or subsequent events^8–11^. Indeed, the “chunking” of daily events is thought to facilitate memory storage, processing, and retrieval, and information that crosses event boundaries is often remembered less well^8,12^. To test this hypothesis, Dunsmoor and colleagues^7^ examined episodic recall of a fear conditioning episode in healthy humans that underwent an extinction procedure in which there was either no event boundary (a normal 10 second inter-trial interval occurred) or an explicit event boundary (subjects were verbally informed of a 10 second break) separating conditioning and extinction. Subjects that experienced the explicit event boundary exhibited greater recall of the conditioning episode (and less durable extinction) relative to subjects that did not experience the event boundary. In other words, subjects that experienced an event boundary between conditioning and extinction exhibited an IED, whereas those that received a continuous procedure did not. The authors suggested that event segmentation of conditioning and extinction promotes the consolidation of the fear memory at the expense of the extinction memory. By this logic, the IED previously reported in both rodents and humans after fear conditioning could be due to event segmentation, rather than a stress-induced extinction impairment.

To test this hypothesis, we compared our standard IED procedure (extinction trials administered 15-min after the last conditioning trial) with a continuous extinction procedure (extinction trials administered 60-sec after the last conditioning trial) after auditory fear conditioning in rats. If event segmentation is necessary for the IED, then we hypothesized that the IED would be eliminated with a continuous extinction procedure. However, in contrast with this hypothesis, the results revealed that eliminating the event boundary between conditioning and extinction did not rescue the IED. Both the continuous and segmented extinction procedures yielded poor long-term extinction, which was manifest as high levels of conditioned freezing to the auditory conditioned stimulus (CS) that did not differ from non-extinguished controls. The most parsimonious interpretation of these results is that the arousal associated with fear conditioning impedes extinction procedures that occur within hours after conditioning.

## Results

**Figure 2:**
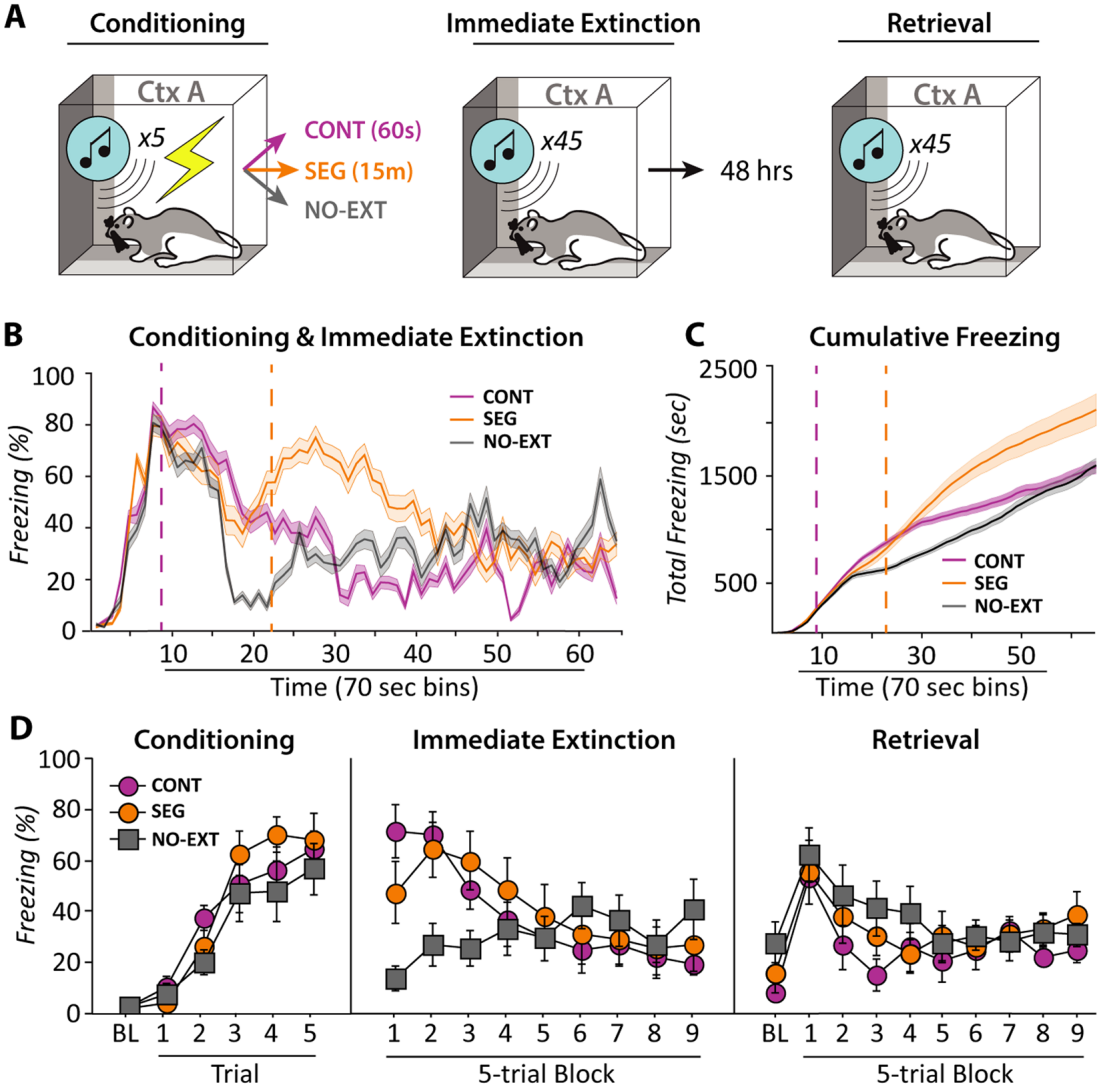
A continuous extinction procedure does not eliminate the IED. (**A**) Illustration of the behavioral groups. Rats were either subjected to a continuous immediate extinction procedure with a normal 60-sec ISI separating conditioning and extinction (continuous, CONT; *n* = 8), a standard immediate extinction procedure with a 15-min event boundary between fear conditioning and extinction (segmented, SEG; *n* = 8), or were not subjected to an extinction procedure (NO-EXT; *n* = 8) immediately following fear conditioning. (**B**) Freezing levels during fear conditioning and immediate extinction of each group plotted in 70 sec bins to match 70 sec inter-trial intervals during extinction. (**C**) Cumulative freezing analysis of acquisition and immediate extinction further confirms behavioral manipulation between groups. Vertical dotted lines denote the beginning of extinction trials for CONT and SEG groups for both B and C. (**D**) All groups had similar acquisition and within-session extinction of fear, as measured by freezing behavior. When tested 48 hr later, both CONT and SEG animals displayed high levels of freezing that were equivalent to NO-EXT animals. Thus, both CONT and SEG animals exhibited an IED. All data are means ± SEMs.

To determine if the IED is a result of event segmentation we devised an experiment (Fig. 2A) in which extinction sessions were presented continuously with fear conditioning (continuous, CONT), after a 15 min break (segmented, SEG), or animals were not extinguished at all immediately following fear conditioning (NO-EXT). Freezing behavior are shown in Fig. 2. All animals exhibited reliable increases in freezing across the conditioning session [main effect of trials: *F*(5,18) = 49.983, *p* < 0.0001] and the levels of freezing did not differ between the groups [trials x group interaction: *F*(10,18) = 0.919, *p* = 0.5195]. During the extinction procedure, all groups exhibited reliable decreases in freezing behavior [main effect of block: *F*(8,18) = 5.154, *p* < 0.0001], as shown in Fig. 2B,D. Cumulative freezing (Fig. 2C) throughout conditioning and immediate extinction indicates, however, that the magnitude of freezing in each of the extinction groups was different over the course of the session [time x group interaction, *F*(2,128) = 91.447, *p* = < 0.0001]. Specifically, rats receiving extinction trials after a 15-min break exhibited higher levels of freezing across the session, largely because the suppression of freezing that accompanies extinction training was delayed by 15-min in this group.

To keep in-line with previous investigations of the IED^1^, all animals received a retention test consisting of another extinction session 48-hours after conditioning. As shown in Fig. 2D, rats in all of the groups exhibited robust fear to the CS, and animals that underwent extinction did not differ from the no-extinction controls [main effect of group: *F*(2,18) = 0.677, *p* = 0.5204; block x group interaction: *F*(16,18) = 0.827, *p* = 0.6533]. There were no sex differences in conditioned freezing during any of the behavioral sessions [conditioning: main effect of sex, *F*(1,18) = 1.728, *p* = 0.2052; immediate extinction: main effect of sex, *F*(1,18) = 2.043, *p* = 0.1701; retention test: main effect of sex, *F*(1,18) = 4.035 *p* = 0.0598], and sex did not interact with the timing of extinction relative to conditioning [conditioning: group x sex interaction, *F*(2,18) = 0.448, *p* = 0.6457; immediate extinction: group x sex interaction: *F*(2,18) = 0.798, *p* = 0.4655; retention test: group x sex interaction, *F*(2,18) = 0.411, *p* = 0.6688].

Although these results suggest that event segmentation does not account for the IED in rodents, confirming the lack of difference between the two extinction groups (i.e., the null hypothesis) is difficult to prove. To address this issue, we calculated the Bayes factor for the first 5-trial block of retrieval testing, as differences in behavioral freezing following immediate and delayed extinction are commonly observed in the first 5 trials of retrieval testing^1^. We found that the null hypothesis (i.e., the IED is not due to event segmentation) was 3.723 times more likely (BF_01_ = 3.723) than the alternative (i.e., the IED is due to event segmentation)^13^. Collectively, these data indicate that the IED occurs with both continuous and segmented extinction procedures, and we provide statistical evidence (BF_01_ > 3) in support of the null hypothesis that event segmentation does not underlie the IED.

## Discussion

Here we explored whether the IED is due to the temporal segmentation produced by a 15-min delay between fear conditioning and extinction^7^. We found that rats that underwent a continuous immediate extinction procedure (with no delay between fear conditioning and extinction) displayed similar within-session extinction to rats that underwent a standard immediate extinction procedure (15-min delay between fear conditioning and extinction). When tested 48 hours later, both groups of animals displayed high levels of freezing that were equivalent to animals that were never extinguished. In other words, animals exhibited an IED independent of whether they experienced an event boundary between conditioning and extinction. Importantly, these findings cannot be contributed to the 48-hour retention interval as we have previously shown that delayed extinction training is retained over 48-hours compared to no-extinction controls^1^. These results contrast with the findings of Dunsmoor and colleagues^7^ insofar as the IED was not overcome with a continuous extinction procedure. We instead point to recent evidence that suggests the IED is a stress-induced impairment.

Considerable work has demonstrated that immediate extinction procedures yield poor long-term extinction memories^1–3,14^. Dunsmoor and colleagues^7^ recently proposed that the IED might be due to a temporal gap (i.e., an event boundary) between fear conditioning and extinction that serves to prioritize the consolidation of the conditioning memory by protecting it from interference from extinction. By this logic, a truly continuous extinction procedure might limit the IED by weakening the fear memory and facilitating the formation of a long-term extinction memory; thus, immediate extinction without an event boundary may be more effective in producing long-term extinction memories than delayed extinction procedures which are not thought to significantly weaken CS-US associations. This echoes an earlier report by Myers and colleagues^4^ who suggested that immediate extinction results in the “unlearning” of the original CS-US association. Consistent with this hypothesis, one study in humans has shown that a continuous extinction procedure blocked the reinstatement of fear-potentiated startle^15^ (i.e., fear did not return after an unsignaled US), suggesting that immediate extinction had weakened the original CS-US associated fear memory. However, other studies in humans using continuous extinction procedures have found strong reinstatement^14,16^ and spontaneous recovery^14^ (i.e., return of fear over time) after immediate extinction as measured by skin conductance responses. Together with the present data, these results suggest that continuous extinction procedures are at least no more, if not less, effective in producing long-term extinction memories relative to both standard immediate and delayed extinction procedures^17–20^.

It is well established that stress influences medial prefrontal cortical circuits known to be involved in extinction learning^5,21^, specifically the infralimbic cortex (IL)^22^. Previous work in rodents has shown that immediate extinction results in diminished c-Fos expression (a correlate of neuronal activity) within the IL, and that the IED can be rescued by IL electrical stimulation^23^. Work from our lab has demonstrated that the IED is associated with impaired IL single-unit activity^22,24^; this decrement, as well as the IED, can be rescued with the β-adrenergic antagonist, propranolol^22,25^. Moreover, the IED can be rescued by pharmacological blockade of either norepinephrine^25^ or corticotrophin releasing factor^26^ within the basolateral nucleus of the amygdala (BLA). By this logic, it seems that impairments in IL activity following fear conditioning underlies the IED, and these impairments may be driven by the BLA. In line with this, stress is known to cause BLA hyperexcitability^27^, and a recent study demonstrated that chronic stress causes increased glutamatergic release from BLA to prefrontal cortical synapses^28^. Thus, we propose that the IED is a result of BLA hyperexcitability which acts to inhibit IL function necessary for extinction learning.

However, it is important to note that the IED has also been observed in paradigms that lack aversive stressors including appetitive conditioning tasks in rats and pigeons^29,30^ and a non-emotional learning task in humans^31^. Nonetheless, the neural circuits mediating the extinction and relapse of appetitive conditioned responses largely overlap with that of aversive conditioning^32,33^. For example, IL lesions enhance the recovery and reinstatement^34^, as well as the renewal^35^, of appetitive conditioned responses. Moreover, the reinstatement of extinguished appetitive conditioned responses in humans is correlated with both amygdala and ventromedial PFC (the human analog of the IL) BOLD responses^36^. Although appetitive conditioning does not use aversive reinforcers, it may nonetheless be stressful. Appetitive conditioning in humans is associated with increased galvanic skin conductance responses and is arousing^36^, and the conditioning and extinction of appetitive responses in rats triggers norepinephrine efflux in the IL^37^. Additionally, appetitive conditioning procedures commonly motivate animals by food deprivation, a stressor. It has previously been shown that neuronal activity in the locus coeruleus (the primary source of norepinephrine^38^) is increased in response to a conditioned food reward in food-deprived rats^39^, but not cats fed *ad libitum*^40^. This suggests that appetitive conditioning and extinction may be stressful in food-deprived animals. Thus, it is possible that the IED observed in both aversive and appetitive conditioning procedures is due to their similar dependence on the IL^33^ and its susceptibility to hyperarousal^5^.

When considering this evidence, it is therefore not surprising that a continuous extinction procedure did not rescue the IED, because stress-evoked impairments in IL function are present immediately after fear conditioning. Moreover, our previous work shows that impairments in the spontaneous firing of IL neurons are maximal within the first 10 minutes after fear conditioning^22^, which is precisely when the initial extinction trials are presented in a continuous extinction procedure. What then explains the lack of an IED in humans undergoing a continuous extinction procedure? Given that the USs used in this study were “highly annoying, but painful” wrist shocks^7^, it is probable that they were not sufficiently stressful to induce an IED. Similarly, the extinction deficit observed in subjects experiencing an event boundary between conditioning and extinction is therefore not likely to be a stress-induced IED. Alternatively, as Dunmoor and colleagues have suggested, the verbal declaration of a transition from conditioning to extinction in their task was sufficient to limit retroactive interference of the conditioning memory by the extinction procedure. It is well established that event segmentation weakens the long-term memory of information which crosses event boundaries^11,41,42^, therefore it is possible that removing the event boundary in humans (continuous extinction) disrupts the fear memory established during conditioning. Moreover, an IED has been reported in a non-emotional predictive learning task in humans, as long as acquisition and extinction of the task occured in the same context^31^. However, it is unclear if an explicit break was inserted between acquisition and extinction. If so, it is likely that these results are indeed due to event segmentation. Interestingly, the rodent hippocampus encodes boundaries between trial and inter-trial intervals^43^, which suggests a neurobiological substrate for event segmentation similar to that in humans^44^. Further work is needed to elucidate whether event segmentation influences memory encoding and retrieval in rodents independently of the stress effects that account for the IED.

Another difference between the current work and that by Dunsmoor and colleagues^7^, is the nature of the conditioning procedure in the two studies. In the human study, subjects received a differential fear conditioning procedure in which visual images from one item category were paired with a wrist shock (CS+, animals or tools; 50% US probability), while images from a second category were never paired with an aversive outcome (CS-, tools or animals, respectively). In the present work, rats were conditioned and extinguished using a single auditory CS. Although a differential fear conditioning procedure^45^, might have more closely mirrored the human work, we chose to use a non-differential conditioning procedure to replicate our previous work on the IED^1,2,22,24,25^. Moreover, in our view, there is no theoretical basis to expect that a differential conditioning procedure would undermine the IED with a continuous extinction procedure in either rats or humans. In contrast, the partial reinforcement schedule in the human study may have made the conditioning and extinction contingencies less discriminable (particularly in the continuous extinction procedure), and this might have encouraged generalization of the conditioning memory and the CS. Indeed, partial reinforcement yields resistance to extinction (i.e., the partial reinforcement extinction effect or PREE^46^) and the verbal instruction in the human study may have provided information that overcame the PREE by reducing generalization from conditioning to extinction. Future work exploring whether partial reinforcement schedules influence the IEDs in rodents would be important to address this issue.

In conclusion, we show that event segmentation is not responsible for the IED previously described in rats^1^. This challenges recent work in humans that suggests that an event boundary between fear conditioning and extinction prioritizes the consolidation of the initial conditioning memory, while protecting it from interference by the subsequent extinction memory^7^. Behavioral and neurobiological evidence in rats instead suggests that the hyperarousal that follows conditioning impairs extinction learning^3^. This is evident by pharmacological blockade of norepinephrine which can rescue both the IED^25^ and IED-associated neural impairments^22^, as well by the fact that delayed extinction can be impaired by a prior stressor^1^. However, translation between human and rodent work remains difficult. Further work is needed to understand rodent’s capacity for event segmentation and how event boundaries impact their learning and memory, as well as the potential role of stress in the IED observed in appetitive conditioning procedures. Moreover, future studies using humans are needed to investigate IED-associated neural deficits, and their effect on memory, that have been observed in rodents. We stress that future investigations using immediate extinction procedures in humans should take into special consideration the potentially confounding effects of event boundaries when designing experiments.

## Methods

### Subjects

Twelve male and twelve female adult Long-Evans rats (Blue-Spruce; 50–57 days old; weighing 200–224 g) were obtained from a commercial supplier (Envigo, Indianapolis, IN). Animals were individually housed in cages upon arrival and kept on a 14:10 h light/dark cycle (lights on at 7:00 AM) with ad libitum access to food and water. Each animal was handled for ~30 sec each day for 5 days prior to experimentation to habituate them to the experimenter. All of the experiments and procedures were conducted during the daytime (light phase) at the same time each day in accordance with the relevant guidelines and regulations approved by the Institutional Animal Care and Use Committee at Texas A&M University.

### Behavioral apparatus and procedure

All behavioral procedures were conducted in a single room containing 8 standard rodent conditioning chambers (30 × 24 × 21 cm; Med Associates, St. Albans, VT) housed in sound-attenuating cabinets. Each chamber was identical and consisted of two aluminum sides, a Plexiglass ceiling and rear wall, and a hinged Plexiglass door. The grid floor contained 19 steel rods (4 mm diameter) spaced 1.5 cm apart that were connected to a shock source and solid-state grid scrambler (Med Associates) for the delivery of footshocks. A loudspeaker mounted on the outside of a grating in one aluminum wall was used to deliver auditory stimuli. Locomotor activity was transduced into an electrical signal by a load cell under the floor of the chamber. Load cell activity was digitized (Threshold Activity Software; Med Associates) and later used to measure freezing behavior. Absences of locomotor activity were only considered freezing if they persisted for at least 1 sec. All procedures were carried out in the same context. The previously described conditioning chambers were illuminated by individual house lights that were mounted within one of the aluminum sides, a pan containing 3% acetic acid was placed under the grid floor, and the entire room was illuminated by ambient red light.

Behavioral testing for this study consisted of fear conditioning immediately followed by an extinction session, and one extinction retrieval session 48 hr after conditioning. Each group contained 4 females and 4 males (*n* = 8) and no animals were excluded from the study. Fear conditioning consisted of a 3-min stimulus-free baseline period, followed by five tone (10 sec, 80 dB, 2 kHz)-shock (2 sec, 1 mA) pairings (60 sec ISI). Immediate extinction sessions (45 trials, 60 sec ISI) proceeded within the same context in a group-dependent manner either continuously (CONT) following conditioning, 15 min after conditioning (segmented, SEG), or animals were not immediately extinguished (NO-EXT). For the CONT animals this meant that the first tone-alone (10 sec, 80 dB, 2 kHz) trial was presented after a normal 60 sec ISI following the last shock offset. Time spent in the chambers were equated for each group (i.e. CONT animals remained in the chamber for 15 min after the last extinction trial). All animals were brought back to the same chambers 48 hrs after conditioning to test extinction memory. This session also consisted of a 3 min baseline followed by 45 tone-alone trials with 60 sec ISIs. All freezing data presented here were measured during CS presentation.

### Data analysis

All data were analyzed with analysis of variance (ANOVA) with variables of sex, group, and trial for conditioning, extinction, and retrieval testing.
